# Impact of the definition of bronchopulmonary dysplasia on neurodevelopmental outcomes

**DOI:** 10.1038/s41598-021-01219-0

**Published:** 2021-11-19

**Authors:** Yea-Seul Han, Sung-Ha Kim, Tae-Jung Sung

**Affiliations:** Department of Pediatrics, Hallym University Medical Center, Kangnam Sacred Heart Hospital, Seoul, Korea

**Keywords:** Diseases, Health care, Medical research, Neurology, Risk factors

## Abstract

Understanding the short and long-term pulmonary and neurologic outcomes of neonates with bronchopulmonary dysplasia (BPD) is important in neonatal care for low-birth-weight infants. Different criteria for BPD may have different associations with long-term outcomes. Currently, two criteria for diagnosing BPD have been proposed by the NIH (2001) and NRN (2019) for preterm infants at a postmenstrual age (PMA) of 36 weeks. We investigated which BPD definition best predicts long-term outcomes. Korean nationwide data for preterm infants born between 24^+0^ and < 32^+0^ weeks gestation from January 2013 to December 2015 were collected. For long-term outcomes, severity based on the NRN criteria was significantly related to neurodevelopmental impairment (NDI) in a univariate analysis after other risk factors were controlled. For the admission rate for respiratory disorder, grade 3 BPD of the NRN criteria had the highest specificity (96%), negative predictive value (86%), and accuracy (83%). For predicting NDI at the 18–24 month follow-up, grade 3 BPD of the NRN criteria had the best specificity (98%), positive (64%) and negative (79%) predictive values, and accuracy (78%) while NIH severe BPD had the highest sensitivity (60%). The NRN definition was more strongly associated with poor 2-year developmental outcomes. BPD diagnosed by NRN definitions might better identify infants at high risk for NDI.

## Introduction

Bronchopulmonary dysplasia (BPD) is a major complication in very low birth weight infants (VLBWIs). More severe BPD is associated with a greater probability of developmental impairment and reduced pulmonary function^1–3^. The medical course of the disease, including the need for rehospitalization and neurodevelopmental disability, is a noted concern among parents and healthcare providers^4^. Understanding how disease severity is associated with developmental delay and readmissions can help guide future research and quality improvement initiatives to reduce the burden of this illness.

BPD was first defined by Northway et al.^5^ over 50 years ago; however, in 2001, the National Institute of Child Health (NIH) revised the categorical definitions of BPD. More recently, inter-center variability in oxygen administration and nasal cannula flow, along with the use of noninvasive ventilation, can bias the NIH classification of BPD presence and severity. In other words, these widely used criteria may not be acceptable for the current clinical situation or the prediction of long-term outcomes of contemporary VLBWIs^6,7^. In accordance with this discrepancy, Jensen et al.^8^ proposed a new definition of BPD that better predicted early childhood morbidity and categorized disease severity according to the mode of respiratory support, regardless of the use of supplemental oxygen. This definition was revised to more accurately predict respiratory readmission and developmental delays in a U.S.-born population.

Hence, we aimed to determine which definition of BPD at a PMA of 36 weeks is most suitable for predicting long-term neonatal outcomes, particularly pulmonary and neurologic outcomes, measured at 18–24 months corrected age (CA), in a multicenter cohort study of VLBWIs based on the nationwide Korean Neonatal Network (KNN) registry.

## Results

**Figure 1:**
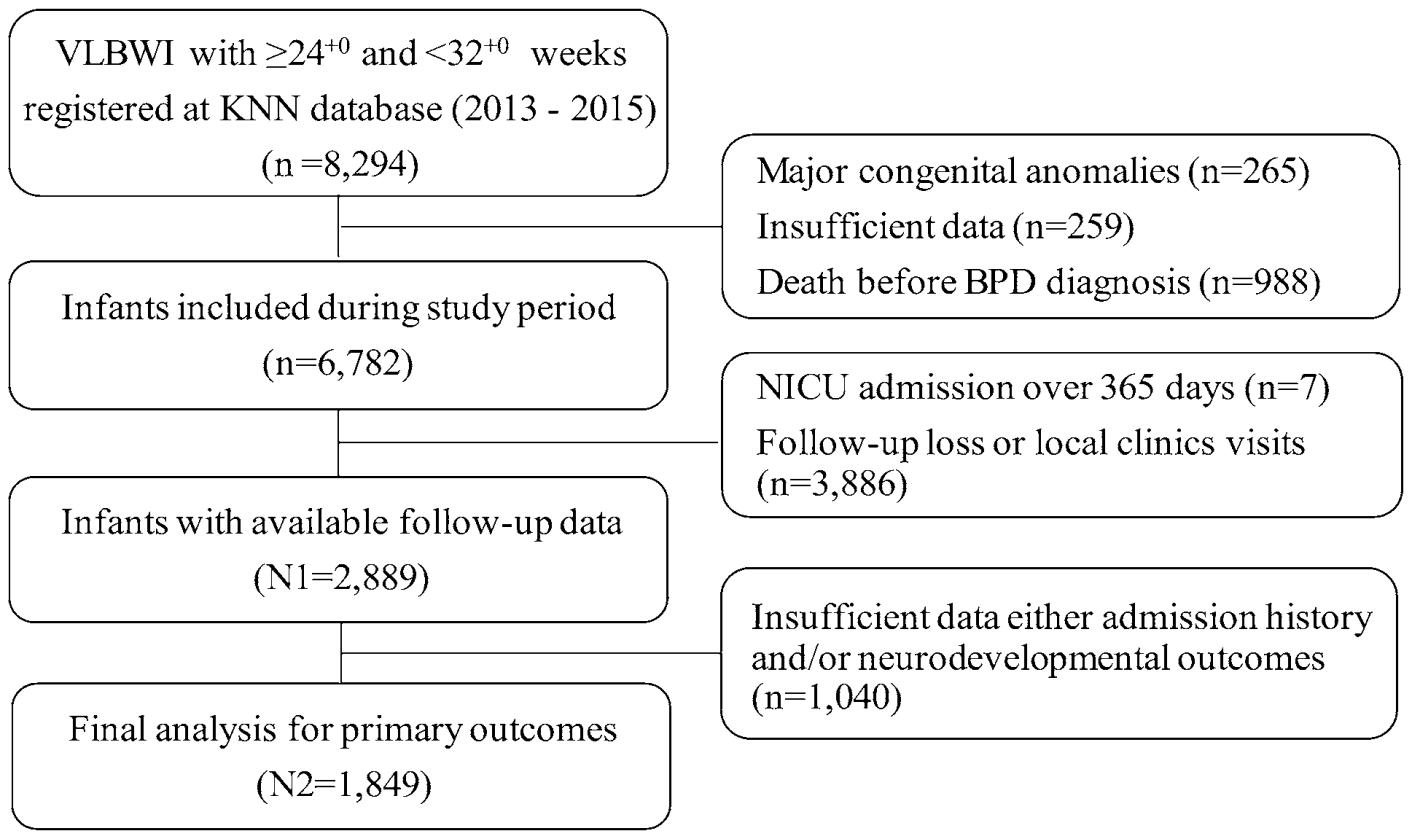
A total of 8294 infants born between 24^+0^ weeks and 31^+6^ weeks GA were enrolled in this study. Of the 8294 patents, 1512 were excluded, as 265 had major congenital anomalies, 259 had insufficient data, and 988 died before BPD diagnosis. At the 18–24 month CA follow-up, 3893 patients were lost to follow-up or visited local clinics. Therefore, 2889 infants were eligible for analysis of respiratory and neurodevelopmental outcomes. Of the 2889 infants, 1040 infants had insufficient follow-up data, and 1849 infants were evaluated in groups in this study.

A total of 8294 infants born between 24^+0^ weeks and 31^+6^ weeks GA were registered with the KNN, After excluding infants, 2889 VLBWIs remained; among them, 1849 (64.0%) had complete follow-up information available at CA 18–24 months, including respiratory readmission and developmental outcomes (Fig. 1). Of those 1849 VLBWIs, 1221 (66.0%) had BPD according to the NIH definition, including 665 infants (36%) with mild BPD, 184 infants (10%) with moderate BPD, and 372 infants (19%) with severe BPD. In contrast 555 infants (30%) had BPD according to the NRN definition, including 186 infants (10%) with grade 1 BPD, 303 infants (16%) with grade 2 BPD, and 66 infants (4%) with grade 3 BPD (Table 1).

**Table 1:** Demographic and baseline characteristics and outcomes of VLBWIs according to different respiratory support approaches (n = 1849).

	NIH criteria					NRN criteria				
	No BPD (n = 628)	Mild (n = 665)	Moderate (n = 184)	Severe (n = 372)	*P*	Grade 0 (n = 2052)	Grade 1 (n = 286)	Grade 2 (n = 439)	Grade 3 (n = 112)	*P*
**Maternal characteristics**										
Maternal age, y	33.0 ± 3.9	32.9 ± 4.1	33.4 ± 4.1	33.1 ± 3.9	0.630	33.0 ± 4.0	33.4 ± 4.2	33.1 ± 3.9	32.9 ± 4.3	0.507
Cesarean section	479(76)	483(73)	130(70)	300(80)	0.013	963(74)	132(71)	245(80)	52(78)	0.049
Ant. corticosteroids	525(83)	552(83)	153(83)	318(85)	0.766	1077(83)	155(83)	257(84)	59(89)	0.557
Maternal DM	61(10)	57(9)	25(13)	27(7)	0.073	118(9)	25(13)	20(6)	6(9)	0.090
Maternal HT	151(24)	92(13)	41(22)	65(17)	< 0.001	243(18)	42(22)	52(17)	12(18)	0.517
Chorioamnionitis *	222(35)	259(38)	81(44)	176(47)	0.001	481(37)	82(44)	144(48)	30(45)	0.003
**Neonatal characteristics**										
GA, weeks	29.5 ± 1.2	27.1 ± 1.7	27.3 ± 1.9	26.5 ± 1.9	< 0.001	28.2 ± 1.9	27.3 ± 1.9	26.5 ± 1.9	26.7 ± 1.8	< 0.001
Birth weight, g	1233.6 ± 199.9	1019.5 ± 226.9	997.6 ± 230.9	881.8 ± 239.8	< 0.001	1123.6 ± 239.3	992.5 ± 234.8	888.3 ± 231.3	857.8 ± 270.4	< 0.001
SGA	99(15)	63(9)	28(15)	93(25)	< 0.001	162(12)	30(16)	70(23)	21(31)	< 0.001
Male	342(54)	339(51)	83(45)	161(43)	0.003	682(52)	84(45)	131(43)	28(42)	0.006
RDS	434(69)	613(92)	170(92)	357(96)	< 0.001	1048(81)	172(92)	288(95)	66(100)	< 0.001
Air leakage	6(1)	14(2)	8(4)	35(9)	< 0.001	20(1)	8(4)	25(8)	10(15)	< 0.001
Pulmonary hemorrhage	3(0.5)	26(3)	17(9)	40(10)	< 0.001	41(2)	23(8)	37(8)	21(19)	< 0.001
Pulmonary hypertension	1(0.2)	20(3)	10(5)	64(17)	< 0.001	21(1)	11(6)	46(15)	17(25)	< 0.001
Postnatal steroid	25(4)	198(30)	71(38)	244(65)	< 0.001	224(17)	71(38)	187(61)	56(84)	< 0.001
PDA, treated	121(19)	300(45)	102(55)	233(62)	< 0.001	422(32)	103(55)	182(60)	49(74)	< 0.001
Sepsis, culture proven	50(8)	128(19)	46(25)	147(39)	< 0.001	179(13)	47(25)	109(36)	36(54)	< 0.001
IVH, grade ≥ 3	8(1)	44(6)	20(10)	57(15)	< 0.001	52(4)	20(11)	44(14)	13(20)	< 0.001
PVL	34(5)	42(6)	27(14)	50(13)	< 0.001	76(6)	27(14)	40(13)	10(15)	< 0.001
NEC, stage ≥ 2	14(2)	39(5)	9(4)	33(9)	< 0.001	53(4)	9(5)	24(8)	9(13)	0.001

*NIH* National Institute of Health, *NRN* Neonatal Research Network, *DM* diabetes mellitus, *HT* hypertension, *GA* gestational age, *SGA* small for GA, *RDS* respiratory distress syndrome, *PDA* patent ductus arteriosus, *IVH* intraventricular hemorrhage, *PVL* periventricular leukomalacia, *NEC* necrotizing enterocolitis.

*Histologic chorioamnionitis for available placentas.

When comparing maternal and infant illness characteristics across BPD definitions, significant differences were observed. Cesarean section was statistically significantly associated with BPD according to both criteria; the highest prevalence of cesarean section was observed in the severe BPD (79%) (*p* < 0.001) and grade II BPD groups (79%) (*p* = 0.014). Histological chorioamnionitis was also significantly associated with both the NIH and NRN definitions of BPD. Maternal hypertensive disorders were significantly different among the NIH criteria groups; the prevalence in the no BPD group was 23%, compared with 13% in the mild BPD group, 20% in the moderate BPD group, and 17% in the severe BPD group (*p* < 0.001). In contrast, in the NRN classification, there were no significant differences among the 4 groups (*p* = 0.527). With respect to neonatal factors, infants with severe or grade III BPD were more likely to have a younger GA and a lower birth weight than infants with no BPD (*p* < 0.001). Other factors, including male sex, RDS, air leakage, pulmonary hemorrhage, pulmonary hypertension, postnatal steroid use, sepsis, IVH, PVL, and NEC, showed significant differences in both criteria. (See online Supplementary Tables S1, S2).

BPD status was significantly associated with unadjusted rates of rehospitalization (≥ 2 times), GMFCS, and NDI, regardless of the criteria used (*p* < 0.001). The frequency of NDI was different between infants with no BPD and those with severe BPD according to the NIH definition (18% and 46% respectively). When the NRN definition was used, 22% of the VLBWIs without BPD had neurodevelopmental disability, compared to 64% of those with grade 3 BPD.

When the NIH definition was used for infants diagnosed with BPD, the severity of BPD did not show any potential for predicting respiratory or neurologic outcomes (Table 2). However, severity based on the NRN definition had a significant relationship with NDI in univariate analysis after other risk factors were controlled. The adjusted odds ratio (OR) for an increase in NDI with BPD compared to no BPD was 1.6 (95% CI 1.1–2.3) for grade 1 (*p* = 0.007), 1.4 (95% CI 1.0–2.0) (*p* = 0.025) for grade 2, and 3.2 for grade 3 (95% CI 1.8–6.0) (*p* < 0.001) (Table 3).

**Table 2:** Multivariable logistic regression analysis model for the relationship between BPD severity at 36 weeks PMA according to NIH criteria and long-term complications.

	No BPD (n = 628)	Mild (n = 665)	OR** (95% CI)	*P*	Moderate (n = 184)	OR** (95% CI)	*P*	Severe (n = 372)	OR** (95% CI)	*P*
Admission for respiratory disorder (≥ 2 times) (%)	178 (13.8)	31 (16.7)	1.0 (0.7– 1.4)	0.915	66 (21.8)	1.1 (0.6–1.7)	0.851	18 (27.3)	1.4 (0.9–2.3)	0.153
GMFCS ≥ 2 (%)	63 (4.9)	21 (11.3)	0.8 (0.4–1.6)	0.574	36 (11.9)	1.3 (0.6–2.8)	0.513	16 (24.2)	1.3 (0.6–2.7)	0.500
NDI (%)*	278 (21.5)	73 (39.2)	0.8 (0.6–1.1)	0.189	128 (42.2)	1.3 (0.9–2.0)	0.179	42 (63.6)	1.4 (0.9–2.1)	0.129

The odds ratios were calculated using “no BPD” as a reference.

*NIH* National Institute of Health, *GMFCS* Gross Motor Function Classification System, *OR* odds ratio, *CI* confidence interval.

*NDI (neurodevelopmental impairment) defined as Bayley composite score < 70(II) or < 85(III) or K-DST < -2 standard deviations.

**OR is adjusted for cesarean section, maternal hypertension, chorioamnionitis, gestational age, birth weight, sex, RDS, air-leakage, pulmonary hemorrhage, pulmonary hypertension, treated PDA, IVH, NEC, sepsis.

**Table 3:** Multiple logistic analysis model of the association between BPD grades at 36 weeks PMA according to NRN criteria had long term complications.

	No BPD (n = 1294)	Grade 1 (n = 186)	OR** (95% CI)	*P*	Grade 2 (n = 303)	OR** (95% CI)	*P*	Grade 3 (n = 66)	OR** (95% CI)	*P*
Admission for respiratory disorder (≥ 2 times) (%)	76 (12.1)	102 (15.3)	1.1 (0.7–1.6)	0.775	31 (16.8)	1.4 (1.0–2.0)	0.081	84 (22.6)	1.9 (1.0– 3.6)	0.054
GMFCS ≥ 2 (%)	26 (4.1)	37 (5.6)	1.5 (0.8–2.7)	0.212	21 (11.4)	1.3 (0.7–2.3)	0.389	52 (14)	3.3 (1.4–7.6)	0.006
NDI (%)*	115 (18.3)	163 (24.5)	1.6 (1.1–2.3)	0.007	71 (38.6)	1.4 (1.0–2.0)	0.025	172 (46.2)	3.2 (1.8–6.0)	< 0.001

The odds ratios were calculated using “no BPD” as a reference.

*NRN* Neonatal Research Network, *GMFCS* Gross Motor Function Classification System, *OR* odds ratio, *CI* confidence interval.

*NDI (neurodevelopmental impairment) defined as Bayley composite score < 70(II) or < 85(III) or K-DST < -2 standard deviations.

**OR is adjusted for cesarean section, maternal hypertension, chorioamnionitis, gestational age, birth weight, sex, RDS, air-leakage, pulmonary hemorrhage, pulmonary hypertension, treated PDA, IVH, NEC, sepsis.

Regarding the rate of admission for respiratory disorder in infants with BPD, among all BPD definition criteria, grade 3 BPD had the highest specificity (96%), negative predictive value (86%), and accuracy (83%). For the prediction of NDI at the 18–24 month follow-up, NRN grade 3 BPD had the best specificity (98%), positive (64%) and negative (79%) predictive value, and accuracy (78%) while NIH severe BPD had the highest sensitivity (60%) (Table 4).

**Table 4:** Predictive values of BPD for admission for respiratory disorder, GMFCS score, and neurodevelopmental impairment at a corrected age of 18–24 months.

	NIH criteria			NRN criteria		
	Mild	Moderate	Severe	Grade1	Grade2	Grade3
**Admission for respiratory disorder**						
Sensitivity	57.3	29.0	52.5	14.8	27.0	9.2
Specificity	49.5	78.3	65.7	87.8	82.5	95.9
Positive predictive value	15.3	16.8	22.6	16.7	21.8	27.3
Negative predictive value	87.9	87.9	87.9	86.2	86.2	86.2
Accuracy	50.6	71.8	63.6	77.5	74.0	83.4
**GMFCS ≥ 2**						
Sensitivity	62.3	46.3	72.4	24.5	39.4	25.2
Specificity	52.1	79.7	68.6	88.1	83.6	96.2
Positive predictive value	5.1	9.2	14.6	9.0	12.0	26.3
Negative predictive value	97.1	97.1	97.1	96.0	96.0	96.0
Accuracy	52.5	78.3	68.9	85.2	81.2	92.6
Neurodevelopmental impairment*						
Sensitivity	58.6	38.2	59.9	20.8	31.5	13.1
Specificity	50.5	81.9	71.9	90.0	85.3	97.7
Positive predictive value	24.5	38.6	46.2	39.2	42.2	63.6
Negative predictive value	81.7	81.7	81.7	78.5	78.5	78.5
Accuracy	52.3	71.9	68.5	73.6	71.6	77.8

The predictive values of BPD were calculated using “no BPD” as a reference.

*BPD* bronchopulmonary dysplasia, *GMFCS* Gross Motor Function Classification System.

*NDI (neurodevelopmental impairment) defined as Bayley composite score < 70(II) or < 85(III) or K-DST < -2 standard deviations.

Follow-up outcomes at 18–24 months CA between subgroups are summarized in online Supplementary Table S3 (10th percentile) (p < 0.001). Growth status including weight (< 10th percentile), height (< 10th percentile), and head circumference (< 10th percentile), showed stepwise increases according to the NIH and NRN groups. Hence, in the severe BPD group, 31% of the children had weights below the 10th percentile (*p* < 0.001), 30% had heights below the 10th percentile (*p* < 0.001), and 36% had head circumferences below the 10th percentile (*p* < 0.001). The same trend was shown for the NRN grade 3 group: 10% had weights below the 10th percentile (*p* < 0.001), 52% had heights below the 10th percentile (*p* < 0.001), and 58% had head circumferences below the 10th percentile (*p* < 0.001).

## Discussion

Compared to previous population-based studies, the strength of this study is that it was a large prospective cohort study that determined the impact of BPD criteria on long-term outcomes especially respiratory and neurologic outcomes. Additionally, our study cohort consisted of infants with a birth weight of less than 1500 g (VLBWIs), who have the highest risk of BPD and related childhood morbidity.

The BPD incidence observed in our cohort seems to be similar to that reported by other investigators; 66% based on NIH criteria vs*.* 30% by NRN criteria^8,9^. This discrepancy of more than double the incidence in the same population may be attributed to the fact that the severity categories used in the current definition of BPD differ according to the clinician’s judgment, especially for distinguishing between mild and moderate or grade 1 or grade 2 BPD. At present, noninvasive ventilation methods are frequently utilized early in the NICU in an attempt to decrease the progression to BPD^10,11^. However, we are not familiar with the definition of BPD that reflects such an approach. For instance, when using the NIH definition, neonatologists may define the severity of BPD differently if a high-flow nasal cannula with FiO_2_ < 30% is being used at the time of diagnosis. This might explain some of the wide variation in BPD incidence and severity at different NICUs.

Hence, in this study we tried to evaluate the best definition for predicting long-term outcomes, including respiratory and neurologic outcomes, in children with BPD at a CA of 2 years. Numerous cohort and epidemiologic reports have indicated that BPD is associated with adverse pulmonary outcomes in early childhood, when comparing infants with BPD to those without BPD; however, those studies also used a variety of definitions and criteria for the condition^12,13^. In our study, univariate analysis showed that the proportion of children with more than two rehospitalizations for respiratory issues increased according to BPD severity or grade, as follows: 15% (mild BPD) vs. moderate (17%) vs. severe (23%); 17% (grade 1) vs. 22% (grade 2) vs. 27% (grade 3). However, after adjustment for confounding factors, there were no statistically significant differences. These results might be due to the subtle deficit in lung function combined with unstudied factors, including environmental factors, such as secondhand smoke inhalation, socioeconomic state, breastfeeding and exposure to viral infection^7^. Another possible reason for this finding might be vaccinations such as monoclonal antibodies of RSV and/or flu vaccination, given that in Korea, premature infants and infants with BPD can be vaccinated free or at very low-cost via public insurance.

In terms of NDI, a stepwise increase was observed with increasing severity, from 22% of the infants with grade 0 (i.e., without BPD) to 64% of those with grade 3 BPD. Similarly, the incidence of NDI increased from 18% of those without BPD to 46% of those with severe BPD. However, after adjusting for confounders, BPD according to the current severity-based definition (the NIH criteria) was not associated with an increased risk of NDI at 18–24 months adjusted age. Moreover, this study showed that the NRN criteria for BPD predicts NDI at 18–24 months better than the classic definition of BPD. A recent study noted that BPD accompanied by invasive mechanical ventilation at 36 weeks PMA strongly predicted NDI (assessed at 2 years), but BPD without invasive mechanical ventilation (i.e., BPD that required only supplemental oxygen) at 36 weeks PMA was not associated with any form of NDI^3^.

We also found that BPD severity was highly correlated with neurodevelopmental morbidity as several previous studies have reported^2,14,15^. As the severity or grade worsened, the probability of complications increased. Infants with BPD tend to have more feeding problems than those without BPD and are more likely to grow poorly and experience frequent and prolonged periods of hypoxemia, which increases the risk of brain injury^16–18^. A difference in childhood growth was also observed in our study. Children with weights below the 10th percentile comprised the major population of the severe BPD and grade 3 groups. The same was true for children with heights below the 10th percentile and head circumferences below the 10th percentile.

There were a few limitations in our study. First, 35.9% of the study group was lost during the follow-up at outpatient clinics, which was a somewhat high proportion. Since Korea offers good medical access through the public insurance system and overall low medical costs, it is easy for the study population to not visit tertiary centers or centers with NICUs. We believe that infants with low BPD severity may be more likely to visit healthcare facilities other than those that were included in our study centers, which were mainly secondary or tertiary centers. This follow-up loss may not have had any effects on our major findings, such as the significant correlation between severity based on the two diagnostic criteria, and the degree to which individual criteria can anticipate long-term outcomes. However, the stepwise correlation between severity and follow-up outcomes may have been biased. Further studies must seriously consider this problem. Studies must try to minimize follow-up loss or analyze the severity and other factors of the lost population and then take the result into account during statistical analyses. The last inherent limitation, associated with the use of registry data, is that management may differ across participating centers. For example, the lack of information concerning a room air test for the classification of BPD severity and although this study reviewed data from 70 NICUs in Korea, the testing ability of those who administer the Bayley scales has not been standardized, which may have caused interobserver discrepancies. Unlike the survival rate and many short-term outcomes, the evaluation of long-term outcomes is challenging due to referral biases, variations in outcome definitions and small sample sizes.

In conclusion, BPD is a complex disease that requires different definitions that better reflect the spectrum of the clinical course. We found that the NRN(2019) definition of BPD was strongly associated with poor 2-year developmental outcomes in VLBWIs. Therefore, we believe there is a need to refine the BPD definition to improve its prognostic value.

## Methods

### Study design and data collection

This prospective cohort study of VLBWIs at 70 neonatal intensive care units (NICUs) was included in the KNN registry. The KNN, which was established by the Korean Society of Neonatology and the Korea Centers for Disease Control and Prevention in 2013, collects nationwide population-based data regarding VLBWIs who have been admitted to the NICU^19^. Participation in the KNN registry was approved by the institutional review board of each participating hospital, including the Hallym University Medical Center (IRB number 2015-05-063), and informed written consent was obtained from parents at enrollment by all of the NICUs participating in the KNN. All of the data were monitored regularly by the KNN data management committee and the KNN ethics committee approved the present study. All methods were performed in accordance with the relevant guidelines and regulations.

VLBWIs born between 24^+0^ and < 32^+0^ weeks gestation from January 2013 to December 2015 were registered with the KNN, and thosewho survived to 1st NICU discharge and were followed through the first 2 years PMA at affiliated outpatient clinics participating in the KNN were collected from the KNN registry. Infants who had life-threatening congenital malformations, died before BPD diagnosis, and were admitted to the NICU for more than 365 days, received follow-up care outside of the KNN system or were missing key study data were excluded.

Maternal characteristics included maternal age, mode of delivery; receipt of one or more doses of antenatal steroids, histological chorioamnionitis, gestational or overt diabetes mellitus, and pregnancy-induced or pre-existing maternal hypertension. Neonatal variables included gestational age, birthweight, small for gestational age (SGA), sex, multiple births, height and head circumference at birth. The evaluated neonatal morbidities were respiratory distress syndrome (RDS), air leakage, defined as pneumothorax, pneumomediastinum; pulmonary hemorrhage, pulmonary hypertension, treatment for patent ductus arteriosus, sepsis, intraventricular hemorrhage (IVH) ≥ grade 3, periventricular leukomalacia (PVL), defined as cystic change on neuroimaging; and necrotizing enterocolitis (NEC) ≥ stage II.

### Definitions of BPD

BPD was defined as oxygen supplementation for ≥ 28 days of life. The study population was classified into four groups according to the respiratory support status at the corrected GA of 36 weeks. First, the clinical definition of BPD adopted by the NIH consensus was used to classify the BPD severity at 36 weeks PMA as no BPD, mild BPD with no oxygen supplementation, moderate BPD with oxygen supplementation < 30%, and severe BPD with oxygen supplementation ≥ 30% and/or need for positive pressure support^20^. Then a new evidence-based definition published by the 2019 Neonatal Research Network (NRN) was applied, that categorized BPD as grade 0 (no BPD); grade 1 (BPD, treated with nasal cannula ≤ 2 L/min); grade 2 (BPD, treated with nasal cannula > 2 L/min or noninvasive positive airway pressure); and grade 3 (BPD, treated with invasive mechanical ventilation)^8^. Noninvasive positive airway pressure that provided positive end-expiratory pressure included high-flow nasal cannula (HFNC), continuous positive airway pressure (CPAP), nasal intermittent positive pressure ventilation (NIPPV), biphasic continuous positive airway pressure, and high flow of air or oxygen. The parameters described below were compared among the four groups based on different BPD criteria.

### Study outcomes

We evaluated infant growth status, including height (cm), weight (g), head circumference (cm), admission for respiratory disorder, and neurodevelopmental outcomes at 18 to 24 months CA. The growth index was measured, and percentiles were obtained in comparison with the growth curve of a term newborn in Korea.

The admission for respiratory disorder (infectious or noninfectious) was defined as two or more rehospitalizations after NICU discharge. During the research period, influenza vaccines and monoclonal antibodies of respiratory syncytial virus were administered each fall if needed. Neurodevelopmental outcomes were assessed as follows: (1) moderate to severe cerebral palsy or Gross Motor Function Classification System (GMFCS) ≥ 2^21^; and (2) neurodevelopmental impairment (NDI). NDI was defined when at least one of following criteria were met: (1) BSID-II Mental Development Index (MDI) or BSID-II Psychomotor Development Index (PDI) score < 70; (2) BSID-III Cognitive Composite (CC) score, Language Composite (LC) score, and Motor Composite (MC) score of < 85; and (3) K-DST score below -2 standard deviations (SD), indicating severe impairment.

All infants were asked prospectively to visit the follow-up clinic and underwent a comprehensive developmental assessment by a multidisciplinary team using the Bayley Scales of Infants Development, Second Edition (BSID-II), or the Bayley Scales of Infants and Toddler Development, Third Edition (BSID-III) and/or Korean Developmental Screening Test (K-DST) for infants and children. The K-DST is a developmental screening tool that has been acknowledged by the Korean Society of Pediatrics for infant and child health screening, and verifies whether Korean infants have met developmental standards in the 6 domains of gross motor, fine motor, cognition, language, social skill, and self-help^22^.

### Statistical analysis

Data from each BPD severity group were compared using one-way ANOVA, the Kruskal–Wallis test for continuous data, the chi-square test or Fisher’s exact test for categorical variables, or z tests with correction for continuity for proportions.

To adjust the association between each BPD definition and outcomes for potential covariates, multivariate logistic regression was performed for each severity group to estimate the odds ratio (OR) with a 95% confidence interval (CI). All regression models were adjusted for the baseline characteristics determined in the univariate analysis. Sensitivity, specificity and positive and negative predictive values were calculated for respiratory disorder, GMFCS score, and NDI using multivariable logistic regression.

The fit of the models was verified with the Hosmer–Lemeshow goodness of-fit test. All analyses were performed using the software package SPSS version 24.0 (SPSS Inc., Chicago, IL, USA). In all analyses, a *p* value less than 0.05 was considered statistically significant.

## Supplementary Information

Supplementary Table 1.

Supplementary Table 2.

Supplementary Table 3.

## Data Availability

The dataset analyzed in this study is not publicly available due to the policy of Research of the Korea Centers for Disease Control and Prevention. However, datasets are available from the corresponding author upon reasonable request.
