# Supplementary material for: Impact of the definition of bronchopulmonary dysplasia on neurodevelopmental outcomes
Source: Sci Rep. 2021 Nov 19;11:22589. doi: 10.1038/s41598-021-01219-0 (PMC8605019; doi:10.1038/s41598-021-01219-0)
Supplement: Supplementary file 1 — Supplementary Table 1. [file 41598_2021_1219_MOESM1_ESM.docx]

Table 1. Demographic and baseline characteristics and short-term outcomes of VLBWIs according to different respiratory supports approaches (n=2,889)

|  | NIH Criteria | | | | |  | NRN Criteria | | | | |
| --- | --- | --- | --- | --- | --- | --- | --- | --- | --- | --- | --- |
|  | No BPD  (n=1,059) | Mild  (n=992) | Moderate  (n=281) | Severe  (n=557) | *P* |  | Grade 0  (n=2,052) | Grade 1  (n=286) | Grade 2  (n=439) | Grade 3  (n=112) | *P* |
| Maternal characteristics | | | | | | | | | | | |
| Maternal age, y | 32.9±4.1 | 32.8±4.3 | 32.7±4.3 | 33.0±4.0 | 0.736 |  | 32.9±4.1 | 32.8±4.3 | 32.9±4.0 | 33.0±3.8 | 0.940 |
| Cesarean section | 829(78) | 729(74) | 193(69) | 440(79) | 0.001 |  | 1559 (76) | 197(69) | 348(79) | 87(78) | 0.014 |
| Ant. corticosteroids | 861(82) | 799(82) | 215(79) | 467(85) | 0.151 |  | 1660 (82) | 220(79) | 365(84) | 97(91) | 0.052 |
| Maternal DM | 95(9) | 86(9) | 33(12) | 35(6) | 0.057 |  | 181(9) | 33(12) | 27(6) | 8(7) | 0.075 |
| Maternal HT | 245(23) | 130(13) | 57(20) | 93(17) | <0.001 |  | 375(18) | 58(20) | 76(17) | 16(14) | 0.527 |
| Chorioamnionitis ^*^ | 270(30) | 321(38) | 108(44) | 206(44) | <0.001 |  | 591(34) | 112(45) | 173(45) | 291(36) | <0.001 |
| Neonatal characteristics | | | | | | | | | | | |
| GA, weeks | 29.5±1.2 | 27.2±1.61 | 27.2±1.8 | 26.7±2.0 | <0.001 |  | 28.4±1.8 | 27.2±1.8 | 26.7±2.0 | 26.7±1.9 | <0.001 |
| Birth weight, g | 1,242.4±193.6 | 1,029.6±222.1 | 1,009.1±239.2 | 909.3±247.3 | <0.001 |  | 1139.5±233.4 | 1005.0 ± 241.1 | 913.2±242.0 | 895.3±265.7 | <0.001 |
| SGA | 158(15) | 87(9) | 42(15) | 124(22) | <0.001 |  | 245(12) | 44(15) | 90(21) | 32(29) | <0.001 |
| Male | 549(52) | 489(49) | 122(43) | 235(42) | <0.001 |  | 1013(49) | 163(57) | 248(57) | 70(63) | <0.001 |
| RDS | 779(74) | 923(93) | 263(94) | 534(96) | <0.001 |  | 1703(83) | 268(94) | 417(95) | 111(99) | <0.001 |
| Surfactant treatment | 783(74) | 947(96) | 271(96) | 541(97) | <0.001 |  | 1731(84) | 276(97) | 424(97) | 111(99) | <0.001 |
| Prophylactic | 286(37) | 191(20) | 84(31) | 118(22) | <0.001 |  | 477(28) | 84(30) | 88(21) | 29(26) | 0.021 |
| Air leakage | 13(1) | 23(2) | 18(6) | 50(9) | <0.001 |  | 36(2) | 18(6) | 33(8) | 17(15) | <0.001 |
| Pulmonary hemorrhage | 7(1) | 34(3) | 22(8) | 59(11) | <0.001 |  | 41(2) | 23(8) | 37(8) | 21(19) | <0.001 |
| Pulmonary hypertension | 2(0.2) | 25(3) | 17(6) | 87(16) | <0.001 |  | 27(1) | 18(6) | 63(14) | 23(21) | <0.001 |
| Postnatal steroid | 43(4) | 294(30) | 119(42) | 354(64) | <0.001 |  | 338(17) | 121(42) | 257(59) | 94(84) | <0.001 |
| PDA, treated | 223(21) | 486(49) | 153(54) | 343(62) | <0.001 |  | 710(35) | 154(54) | 264(60) | 77(69) | <0.001 |
| Sepsis, culture proven | 99(9) | 210(21) | 73(26) | 226(41) | <0.001 |  | 31(15) | 75(26) | 166(38) | 57(51) | <0.001 |
| IVH, grade ≥3 | 13(1) | 64(7) | 33912) | 84(15) | <0.001 |  | 77(4) | 33(12) | 59(13) | 25(22) | <0.001 |
| PVL | 559(5) | 63(6) | 38(14) | 81(15) | <0.001 |  | 118(6) | 39(14) | 61(14) | 19(17) | <0.001 |
| NEC, stage ≥2 | 21(2) | 50(5) | 12(4) | 51(9) | <0.001 |  | 71(4) | 12(4) | 39(9) | 12(11) | <0.001 |
| Death after 1^st^ discharge | 5(0.5) | 3(0.3) | 3(0.7) | 10(2) | 0.005 |  | 8(0.4) | 2(0.7) | 5(1.1) | 5(4.5) | <0.001 |

* Histologic chorioamnionitis for available placentas

Abbreviations: NIH, National Institute of Health; NRN, Neonatal Research Network; DM, diabetes mellitus; HT, hypertension; GA, gestational age; SGA, small for GA; RDS, respiratory distress syndrome; PDA, patent ductus arteriosus; IVH, intraventricular hemorrhage; PVL, periventricular leukomalacia; NEC, necrotizing enterocolitis.
