# Supplementary material for: Impact of the definition of bronchopulmonary dysplasia on neurodevelopmental outcomes
Source: Sci Rep. 2021 Nov 19;11:22589. doi: 10.1038/s41598-021-01219-0 (PMC8605019; doi:10.1038/s41598-021-01219-0)
Supplement: Supplementary file 2 — Supplementary Table 2. [file 41598_2021_1219_MOESM2_ESM.docx]

Table 2.Proportion or mean comparison of infants who were lost to follow up and those who were examined

| Variables | Lost to follow-up*(n=1,040) | Examined*(n=1,849) | *P* |
| --- | --- | --- | --- |
| Maternal age, year | 32.600 | 33.026 | 0.007 |
| Cesarean section | 0.768 | 0.752 | 0.376 |
| Maternal diabetes mellitus | 0.077 | 0.091 | 0.207 |
| Maternal hypertension | 0.169 | 0.189 | 0.209 |
| NIH criteria (with BPD) | 0.586 | 0.660 | <0.001 |
| NRN criteria (with BPD) | 0.271 | 0.329 | 0.001 |
| GA, weeks | 28.192 | 27.787 | <0.001 |
| Birth weight, g | 1118.065 | 1062.353 | <0.001 |
| SGA | 0.123 | 0.153 | 0.031 |
| Sex, male | 0.548 | 0.500 | 0.014 |
| RDS | 0.889 | 0.851 | 0.005 |
| Air leakage | 0.039 | 0.034 | 0.524 |
| Pulmonary hemorrhage | 0.035 | 0.047 | 0.153 |
| Pulmonary hypertension | 0.035 | 0.051 | 0.047 |
| Postnatal steroids | 0.262 | 0.291 | 0.100 |
| Pulmonary ductus arteriosus | 0.432 | 0.409 | 0.247 |
| Sepsis | 0.228 | 0.201 | 0.094 |
| IVH | 0.063 | 0.070 | 0.502 |
| *Proportion or mean  Abbreviations: NIH, National Institute of Health; NRN, Neonatal Research Network; BPD, bronchopulmonary dysplasia; GA, gestational age; SGA, small for GA; RDS, respiratory distress syndrome; IVH, intraventricular hemorrhage | | | |
