# Supplementary material for: Impact of the definition of bronchopulmonary dysplasia on neurodevelopmental outcomes
Source: Sci Rep. 2021 Nov 19;11:22589. doi: 10.1038/s41598-021-01219-0 (PMC8605019; doi:10.1038/s41598-021-01219-0)
Supplement: Supplementary file 3 — Supplementary Table 3. [file 41598_2021_1219_MOESM3_ESM.docx]

Table 3. Neonatal follow-up outcomes at 18 - 24 months corrected age according to the NICHD and AJRCCM definition (n=1,849)

|  | NIH criteria | | | | |  | NRN criteria | | | | |
| --- | --- | --- | --- | --- | --- | --- | --- | --- | --- | --- | --- |
| Characteristics | No BPD  (n=628) | Mild  (n=665) | Moderate  (n=184) | Severe  (n=372) | *P* |  | No BPD  (n=1,294) | Grade1  (n=186) | Grade2  (n=303) | Grade3  (n=66) | *P* |
| Weight, <10% 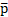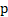 | 68(10.8) | 101(15.2) | 39(21.1) | 115(30.9) | <0.001 |  | 168(12.9) | 39(20.9) | 78(25.7) | 34(51.5) | <0.001 |
| Height, <10% | 94(14.9) | 122(18.3) | 42(22.8) | 112(30.1) | <0.001 |  | 207(15.9) | 43(23) | 78(25.7) | 34(51.5) | <0.001 |
| Head circumference, <10% | 83(13.2) | 100(15.3) | 46(25.0) | 133(35.7) | <0.001 |  | 183(14.1) | 47(25.3) | 98(32.3) | 38(57.6) | <0.001 |
| Admission for respiratory disorder (≥ 2 times) (%) | 76(12.1) | 102(15.3) | 31(16.8) | 84(22.6) | <0.001 |  | 178(13.8) | 31(16.7) | 66(21.8) | 18(27.3) | <0.001 |
| GMFCS ≥2 (%) | 26(4.1) | 37(5.6) | 21(11.4) | 52(14) | <0.001 |  | 63(4.9) | 21(11.3) | 36(11.9) | 16(24.2) | <0.001 |
| NDI (%)* | 115(18.3) | 163(24.5) | 71(38.6) | 172(46.2) | <0.001 |  | 278(21.5) | 73(39.2) | 128(42.2) | 42(63.6) | <0.001 |

Abbreviations: NIH, National Institute of Health;NRN, Neonatal Research Network; GMFCS, Gross Motor Function Classification System; NDI, neurodevelopmental impairment

*NDI(neurodevelopmental impairment) defined as Bayley composite score <70(II) or <85(III) or K-DST < -2 standard deviations
